# Anchoring Stealth Amine Biomarkers via Coupled Chemical Activation for Surface Immobilization

**DOI:** 10.1021/acs.langmuir.5c06581

**Published:** 2026-04-07

**Authors:** Jinyoung Choi, Sehyun Park, Jaejun Lee, Vladimir V. Tsukruk

**Affiliations:** School of Materials Science and Engineering, 1372 Georgia Institute of Technology, Atlanta, Georgia 30332, United States

## Abstract

Detection of low-activity tertiary amine-containing biomarkers such as *N*,*N*-dimethyltryptamine remains a significant challenge due to the inherent electrochemical inertness of the functional groups. Thus, we demonstrate an integrated functionalization strategy combining selective chemical activation with surface-confined transamidation chemistry to enable the comprehensive chemical activation of the analyte and selective binding to modified gold surfaces. This step facilitates an effective surface immobilization strategy that selectively converts inert indole-based tertiary amines into reactive, surface-bondable derivatives suitable for potential electronic biosensing applications. Our approach integrates the selective oxidation of tertiary amines to N-oxides, followed by the Polonovski activation to form reactive tertiary amides in solution. This stage is further coupled with the covalent immobilization of the activated amides onto an −NH_2_-terminated self-assembled monolayer via transition-metal-free transamidation surface chemistry. Surface analysis revealed modified topology and properties. The high grafting density of the adsorbed layer, along with the corresponding elemental analyses, confirms the successful immobilization of the activated derivatives in contrast to nonactivated molecules. This surface reactivity triggering demonstrates the transformation of chemically inert tertiary amines in an important class of drugs into surface-bound reactive species with high selectivity in surface adsorption, establishing a foundation toward label-free selective binding for real-time bioelectronic monitoring for health diagnostic and forensic applications.

## Graphical abstract

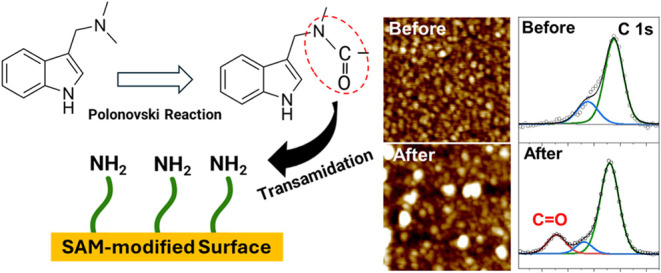

## Introduction

The selective detection of specific chemical and biological analytes in complex environments remains a major challenge in analytical chemistry, particularly when targeting structurally similar or low-reactivity compounds.^1,2^ Among them, low-profile psychedelic biomarkers such as *N*,*N*-dimethyltryptamine (DMT) and related analytes from a class of tryptamine alkaloids, remain largely “invisible” for conventional detection schemes, despite significant implications in neurochemistry research, psychiatric diagnostics, and forensic science.^3^ Direct electrochemical detection of these biomarkers presents formidable challenges due to the inherent inertness of the tertiary amine functionality. The absence of easily oxidizable or reducible groups in the side chain makes conventional electrochemical sensory approaches ineffective at clinically relevant concentrations.^4^

Furthermore, the high degree of structural similarity between DMT analytes and other endogenous tryptamines creates stringent selectivity requirements for diagnostic applications.^5^ Consequently, the development of selective and mild chemical methodologies that can convert the inert tertiary amine into a reactive moiety suitable for biosensor integration remains a critical challenge.^6^ Moreover, current analytical testing approaches often require lengthy pretreatment protocols, high-temperature reactions, or high-cost analysis equipment, limiting their practical applicability in real-time biosensing contexts and in vivo monitoring scenarios.^7,8^

Overcoming these barriers requires systems integrating chemical reactivity with sophisticated surface engineering of sensory electrodes.^9^ For instance, researchers reported the utilization of molecularly imprinted polymers on substrates, which exploited both redox activity and steric exclusion to detect DMT molecules via monitoring electrochemiluminescence modulation.^4^ On the other hand, functionalized self-assembled monolayers (SAMs) can serve as a framework for immobilizing diverse activated biomarkers onto sensor surfaces with precise spatial control.^10,11^ Critically, SAMs enable the creation of chemically tailored interfaces through on-demand selection of terminal functional groups (such as amino, carboxyl, hydroxyl, or alkyl), allowing the customization of surface properties for specific biorecognition tasks.^12,13^

In bioanalytical sensing approaches, SAM-modified surfaces serve three essential roles. First, they establish a biorecognition layer by presenting functional surface groups such as amino (−NH_2_), carboxyl (−COOH), or thiol (−SH) groups that can covalently immobilize target molecules or recognition elements.^14,15^ This immobilization occurs with high efficiency and selectivity, minimizing the nonspecific binding and false signals. Second, SAMs provide electrochemical accessibility, allowing an efficient electron transfer between the surface-bound biomarkers and the underlying electrodes, which is a requirement for the electrochemical and potentiometric sensing modalities in bioelectronic systems.^16^ For instance, amino-terminated SAMs on gold surfaces have proven to offer superior electron transfer kinetics compared to bare gold, enabling voltametric detection of the analytes bound to the surface.^17^ Finally, SAMs modulate interfacial properties quantifiably. Biomarker binding alters surface charge, hydrophilicity, and dielectric properties, changing the electrochemical impedance proportionally, which is essential for quantitative monitoring. Indeed, a selective biomolecular sensing platform based on SAMs and field-effect electronics has been demonstrated for various proteins with excellent sensitivity and efficiency.^18,19^

Looking back to the tertiary amine-containing compounds, a classical, yet underexploited strategy for tertiary amine functionalization is the Polonovski reaction, which transforms tertiary amines into the corresponding secondary amides through a multistep oxidative process.^20^ In this sequence, a tertiary amine is first oxidized to the corresponding N-oxide using hydrogen peroxide, which is a mild and readily available oxidant. This N-oxide intermediate, while retaining the basic indole scaffold intact, possesses dramatically altered reactivity compared with the parent amine. Subsequently, treatment of the N-oxide with acetic anhydride (Ac_2_O) triggers a rearrangement reaction wherein an iminium intermediate is generated through the loss of an acetyl group from the anhydride. This iminium is then intercepted by in situ-generated nucleophiles or rearranges to form a tertiary amide product, effectively transforming the tertiary amine into a more reactive amide functionality.

The appeal of the Polonovski reaction for prospective biosensing applications targeting tertiary amines lies in its remarkable selectivity. Unlike many amine-based transformations, the N-oxidation occurs exclusively for tertiary amines and does not proceed with primary or secondary amines or other nitrogen-containing functional groups.^20^ This binding selectivity is further reinforced in the subsequent Polonovski rearrangement, which is specific to the N-oxide intermediate and does not occur with competing functional groups. The combination of these transformations provides a high degree of reactive specificity while operating under ambient conditions without requiring toxic transition metals or harsh reaction conditions. These features are particularly valuable when the target analyte must be distinguished from closely related impurities or metabolites in complex biological matrices. Furthermore, recently, a transition-metal-free transamidation reaction has been suggested as a strategy for the covalent immobilization of biomarkers onto amino-terminated self-assembled monolayers.^21,22^ Notably, the transamidation exhibits high chemoselectivity for primary amines over secondary amines and other nucleophiles, ensuring selective coupling even in the presence of competing functional groups. This convergent coupling strategy overcomes the limitations of traditional carbodiimide- or peptide-coupling methods.

Here, we propose the integration of the Polonovski reaction, transition-metal-free transamidation, and SAMs electrode surface functionalization to establish a potential platform for selective activation and surface immobilization of the inert tertiary amine biomarkers (Figure 1).

**Figure 1:**
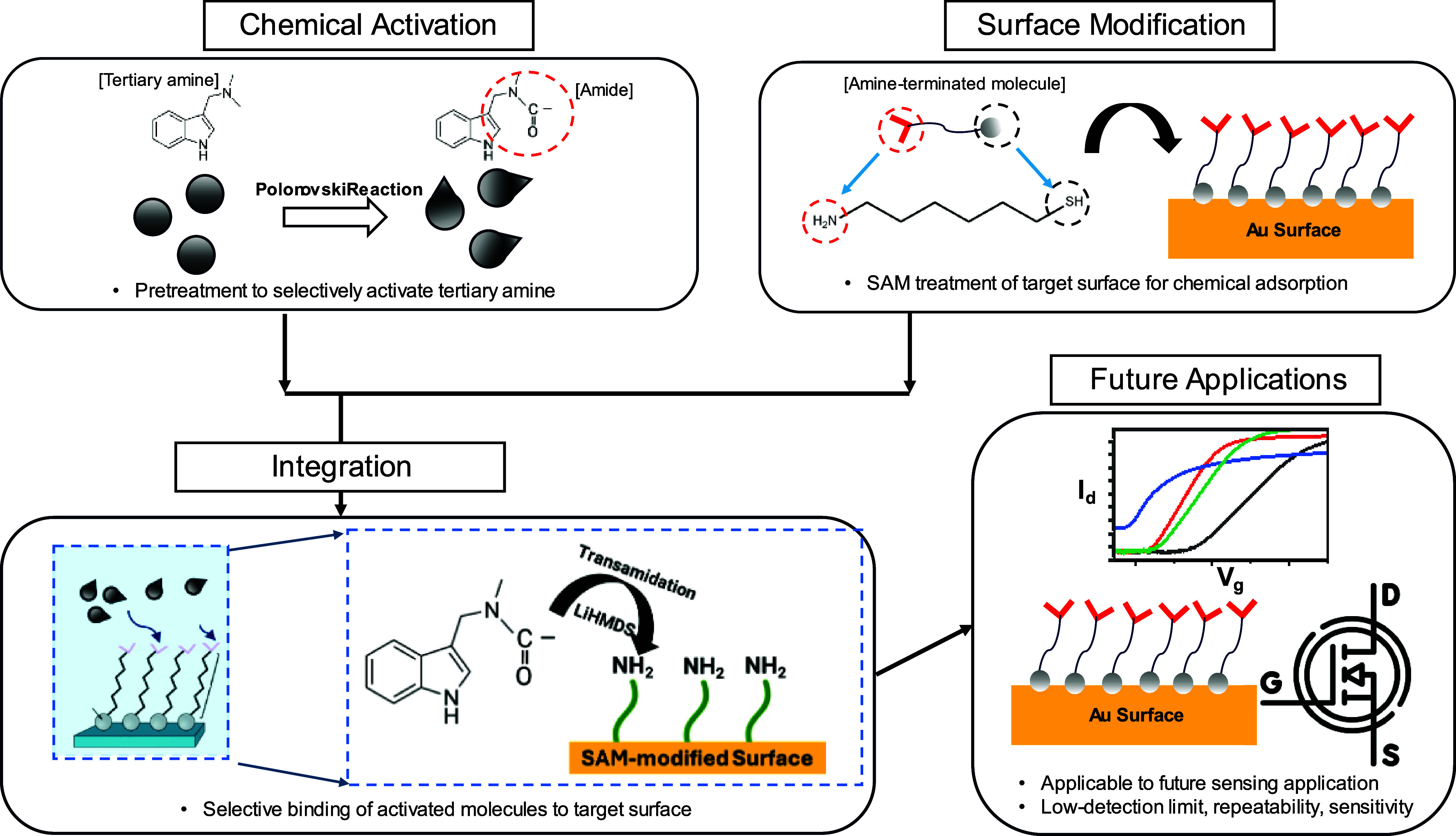
Schematics of selective activation of tertiary amines and integration with a SAM surface. The inert reaction analyte is first chemically activated, while the target surface is chemically modified with SAMs, followed by the integration of the activated analytes to the modified surface via transamidation reaction, for future FET sensing.

First, we selectively activate the tertiary amine biomarkers to N-oxide and finally to amides using the Polonovski reaction while fabricating a functionalized surface by binding primary amine group molecules to the gold surface. Second, the transamidation reaction between the activated analytes and the modified surface establishes a firm, selective bonding between the analytes and the surface, which can serve as a direct sensing surface for future applications by building a pathway to a functional biorecognition interface. This pathway facilitates the integration into field-effect transistor (FET)-based biosensors, where the immobilized layer transduces chemical binding into electronic signals for biosensor applications capable of label-free detection in biological fluids.^23,24^

## Results and Discussion

### Selective Chemical Activation of Tertiary Amines

It is important to note that in this study, we primarily used gramine as a model compound for DMT and as the model tertiary amine. Gramine contains an indole ring and a tertiary amine group, making it very similar in structure to DMT. Meanwhile, tryptamine, which has a similar indole-based structure but contains a primary amine rather than a tertiary amine, was used as a comparison compound. This approach was necessary because drug-related legal restrictions practically prohibit direct use of DMT in laboratories (Figures 1 and S1).

Figure 2 describes the procedures for selectively transforming indole-based tertiary amine biomarkers and binding the activated molecule to a SAM-modified surface. A sequential three-stage process has been designed to convert the chemically inert tertiary amine into a surface-bound biorecognition element. In the initial stage, gramine undergoes selective oxidation to form the corresponding N-oxide intermediate upon treatment with hydrogen peroxide (H_2_O_2_) in acetone or other solvents (Figure S2). This mild oxidative transformation selectively targets the tertiary amine functionality while leaving the indole core intact, generating a reactive N-oxide species (N^+^–O^–^) that serves as a key intermediate for subsequent rearrangement chemistry.

**Figure 2:**
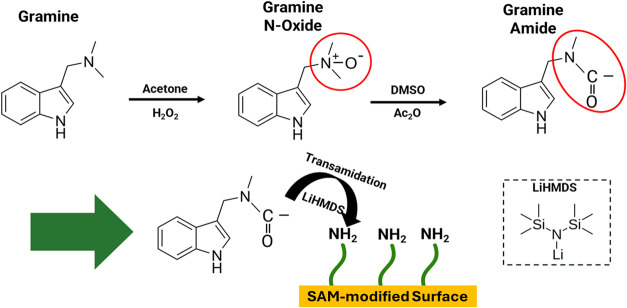
Multistep chemical activation and surface immobilization of gramine. Gramine undergoes selective H_2_O_2_ oxidation in acetone to form the reactive N^+^–O^–^ intermediate, followed by Polonovski rearrangement with Ac_2_O in DMSO to generate activated gramine amide. The gramine amide is then immobilized onto amino-terminated SAM-modified surfaces via transition-metal-free transamidation with LiHMDS, resulting in selective N–C bond cleavage and covalent attachment to surface-bound amine groups (−NH_2_).

The N-oxide intermediate is subsequently subjected to the Polonovski rearrangement by treatment with acetic anhydride (Ac_2_O), wherein an iminium intermediate is formed through the acetyl group transfer from the anhydride (Figure 2). The rearrangement of this iminium species yields the corresponding gramine amide product, in which the tertiary amine has been formally converted to a tertiary amide bearing a carbonyl group directly attached to the nitrogen atom. This activated amide derivative now possesses significantly enhanced electrophilicity at the amide carbonyl, making it susceptible to nucleophilic attack.

In the final stage, the gramine amide is immobilized onto the SAM-modified gold surface through a transition-metal-free transamidation reaction (Figure 2).

When the activated gramine amide is brought into contact with the surface-bound primary amine groups (−NH_2_) in the presence of lithium hexamethyldisilazide (LiHMDS) as a strong base and nucleophile, a selective N–C bond cleavage of the amide carbonyl occurs at the interface. The primary amine nucleophile attacks the amide carbonyl carbon, triggering a rearrangement that results in the formation of a new N–C­(O)– gramine bond covalently linking the bioactive indole alkaloid to the biosensor surface. This transamidation process exhibits an exceptional selectivity for amino-functionalized surfaces, ensuring that the immobilization occurs exclusively at the intended SAMs interface while avoiding nonspecific side reactions, as will be discussed below.

Following this route, the conversion of gramine to N-oxides via H_2_O_2_ was conducted first to follow the chemical changes (Figures 3 and S2–S4). A schematic illustration of the transformation of gramine (top) to the N-oxide product (bottom) is shown in Figure 3a, highlighting the formal conversion of the tertiary amine to the N^+^–O^–^ zwitterionic species. The successful formation of gramine N-oxide was mainly confirmed through ^1^H NMR spectroscopy, which provides diagnostic chemical shift evidence for the oxidative transformation of the tertiary amine to its corresponding N-oxide (Figure 3b).

**Figure 3:**
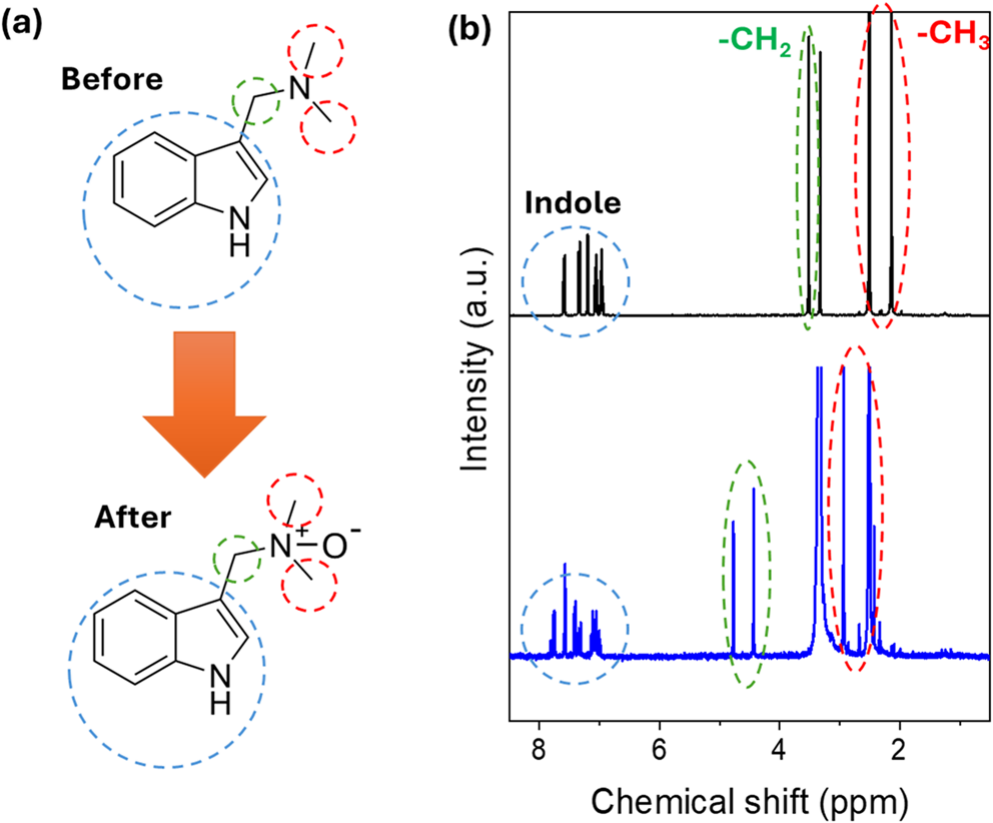
Confirmation of gramine N-oxide formation by NMR: (a) Schematic showing gramine before and after N-oxide transformation. (b) Overlaid ^1^H NMR spectra highlighting the diagnostic 0.8–1.2 ppm downfield shift of *N*-methyl and *N*-methylene protons, distinguished using dashed ovals of different colors. The upper NMR spectrum represents the gramine starting material, and the lower spectrum represents the gramine N-oxide product.

The most diagnostic evidence of successful N-oxide formation is the pronounced downfield shift of magnitude 0.8–1.2 ppm for the *N*-methyl and *N*-methylene protons.^25^ In the starting gramine material, the N-bound methyl groups exhibit characteristic chemical shifts typical of −CH_3_ tertiary amine protons at approximately 2 to 3 ppm, while indole-bound −CH_2_ protons are positioned at 3.5 ppm.

Upon oxidation to form the N-oxide, these same protons experience a substantial deshielding effect resulting from the electron-withdrawing N-oxide functionality, leading to the aforementioned clear downfield shift. Here, benzylic CH_2_ signals appear as a doublet at 4.43 and 4.75 ppm after the N-oxide is formed (Figure 3b). This diastereotopic splitting is characteristic of N-oxide formation, where the electron-withdrawing N^+^–O^–^ group creates a stereogenic center on nitrogen, rendering the two benzylic protons magnetically nonequivalent.^26,27^ At the same time, the region from 7 to 8 ppm corresponds to the indole protons and remains essentially unchanged, confirming that the oxidation has occurred exclusively at the amine functionality while the indole core remains intact.^28^

Overall, the observed chemical shift changes provide a strong confirmation that the selective oxidation reaction proceeded with a high fidelity, generating the reactive N-oxide intermediate required for the subsequent Polonovski rearrangement chemistry.^20,29^ In contrast, when tryptamine, an indole-based primary amine lacking the tertiary amine group compared to gramine, was subjected to identical N-oxide formation conditions, no significant downfield shift of the N-bound or indole-ring-bound −CH_2_ protons was observed in the ^1^H NMR spectrum, but instead the hydrogen peroxide underwent side reactions with the acetone solvent much more than did the tryptamine (Figure S4). This absence of characteristic N-oxide formation signatures for tryptamine confirms the selectivity of the hydrogen peroxide oxidation toward tertiary amines and demonstrates that secondary amines do not undergo N-oxide formation under the applied reaction conditions. This differential reactivity provides a direct experimental validation that the Polonovski activation pathway is selective for tertiary amine structures while remaining inert to secondary amine analogues, thereby establishing the structural selectivity.

To complement the NMR-based structural characterization, measurements of surface potential and solution conductivity were performed using an instrument incorporating dynamic light scattering (DLS) to provide real-time electrochemical confirmation of N-oxide formation via comparison of surface potential measurements for pristine gramine and the gramine N-oxide product (Figure 4).

**Figure 4:**
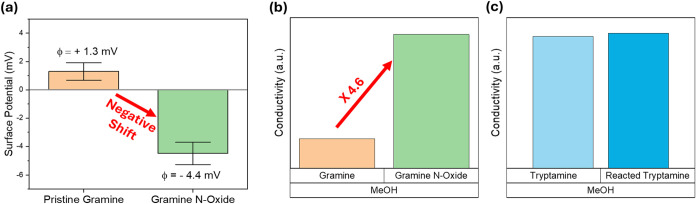
Confirmation of gramine N-oxide formation via ζ-potential and solution conductivity measurements. (a) Surface potential box plot comparing the surface potentials of pristine gramine and gramine N-oxide, showing a pronounced negative shift of −5.7 mV, indicating successful formation of the zwitterionic N^+^–O^–^ species. (b) Results of solution conductivity measurements in methanol showing a dramatic increase in conductivity following N-oxide formation, and (c) not showing a notable conductivity change for the tryptamine comparison group after the same treatment.

Apparently, ζ-potential analysis revealed a pronounced negative shift in surface potential upon N-oxide formation, with the measured surface potential decreasing from +1.3 mV for pristine gramine to −4.4 mV for the gramine N-oxide product. This large 5.7 mV shift in surface potential changes the sign alternation and is consistent with the formation of the zwitterionic N^+^–O^–^ species, wherein the negatively charged oxygen atom creates a localized electrostatic environment that is measurably reflected in the bulk solution charge characteristics.^30^ Complementary solution conductivity measurements conducted in methanol solvent were also made, demonstrating a remarkable up to ×4.6-fold increase in the relative solution conductivity following N-oxide formation in methanol (Figure 4b). Similar increasing trends were also confirmed when EtOH or DMSO/H_2_O were used as solvents (Figure S5).

The zwitterionic N^+^–O^–^ structure possesses a large dipole moment, which can enhance the solvation and dissociation of background electrolytes, contributing additional mobile ions that amplify the measured conductivity.^31^ Importantly, this marked conductivity was confirmed as selective to tertiary amines bearing the gramine structure. As expected, treatment of tryptamine under identical N-oxide formation conditions yielded no significant change in solution conductivity (Figure 4c).

Next, the Polonovski rearrangement of gramine N-oxide to gramine amide was conducted and confirmed through complementary Fourier-transform infrared spectroscopy (FTIR) and NMR spectroscopic analysis (Figure 5). The FTIR spectra show the gramine N-oxide, the characteristic N–H stretching band of the indole core at approximately 3300 cm^–1^, alongside broad C–H stretching features in the 2800–3000 cm^–1^ region (Figure 5b,c).^32^ Most importantly, the gramine amide spectrum displays a prominent carbonyl C=O group absorption peak at approximately 1650 cm^–1^, which, in contrast, is not observed for the gramine N-oxide before the reaction, directly confirming the formation of an amide functional group. This selective appearance of the C=O absorption in the product spectrum, combined with its complete absence in both the gramine starting material and gramine N-oxide intermediate, confirms a successful tertiary amide formation.

**Figure 5:**
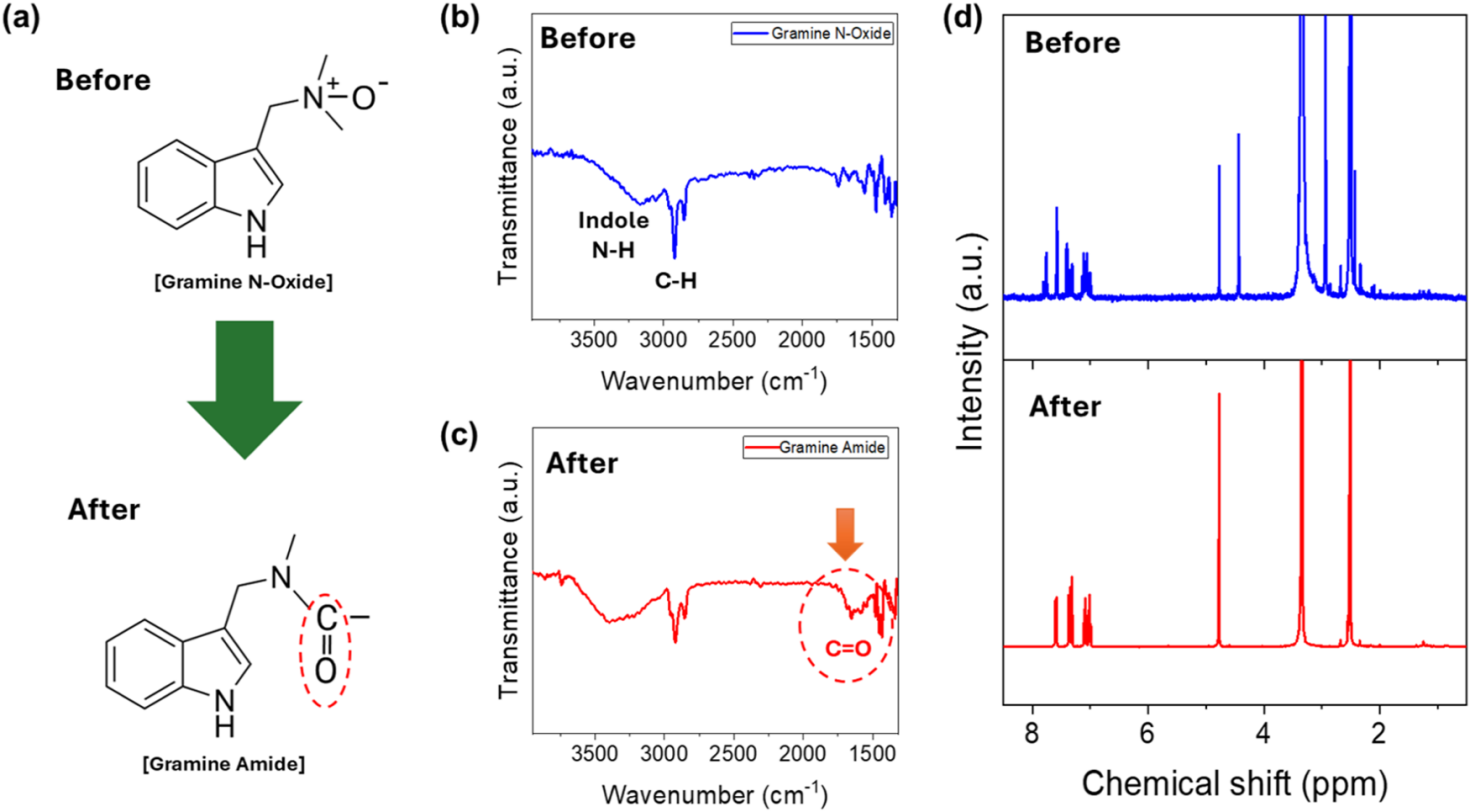
Confirmation of gramine amide formation. (a) Chemical structures showing the conversion of gramine N-oxide to gramine amide, with the amide C=O bond formed after the reaction marked by a red dotted circle. FTIR spectra of (b) gramine N-oxide and (c) gramine amide product. Both plots show characteristic indole N–H stretching (∼3300 cm^–1^) and C–H stretching features (2800–3000 cm^–1^). After the reaction, the gramine amide spectrum displays a characteristic carbonyl absorption peak at ∼1650 cm^–1^ (red circle), confirming amide formation. (d) Comparison of ^1^H NMR spectra for gramine N-oxide (upper) and the gramine amide product (lower).

In addition, NMR was conducted again to compare the initial gramine N-oxide and gramine amide products (Figure 5d). Compared to the NMR spectrum for the N-oxide, the gramine amide spectrum reveals a single benzylic CH_2_ peak at 4.77 ppm, indicating the loss of diastereotopic splitting and consistent with amide formation where the stereogenic center is no longer present.^26,27^ The preserved indole aromatic protons at 7–8 ppm again demonstrate that the indole core remains structurally intact throughout the rearrangement, with the transformation occurring exclusively at the side chain tertiary amine. Overall, the diagnostic benzylic CH_2_ chemical shift changes and the loss of diastereotopic splitting confirm the successful conversion of gramine N-oxide to gramine amide, validating this route as an effective activation strategy for tertiary amine conversion to reactive amide intermediates.

### Surface Functionalization and Targeted Molecular Anchoring

Simultaneously, we prepared SAM-modified gold surfaces suitable for subsequent gramine amide immobilization (Figures 6 and S6). Figure 6a illustrates the preparation strategy, wherein an amino-functionalized thiol compound, 6-amino-1-hexanethiol, is adsorbed onto a pristine gold surface, forming a densely packed monolayer through the strong gold–sulfur coordinative interaction.^33,34^

**Figure 6:**
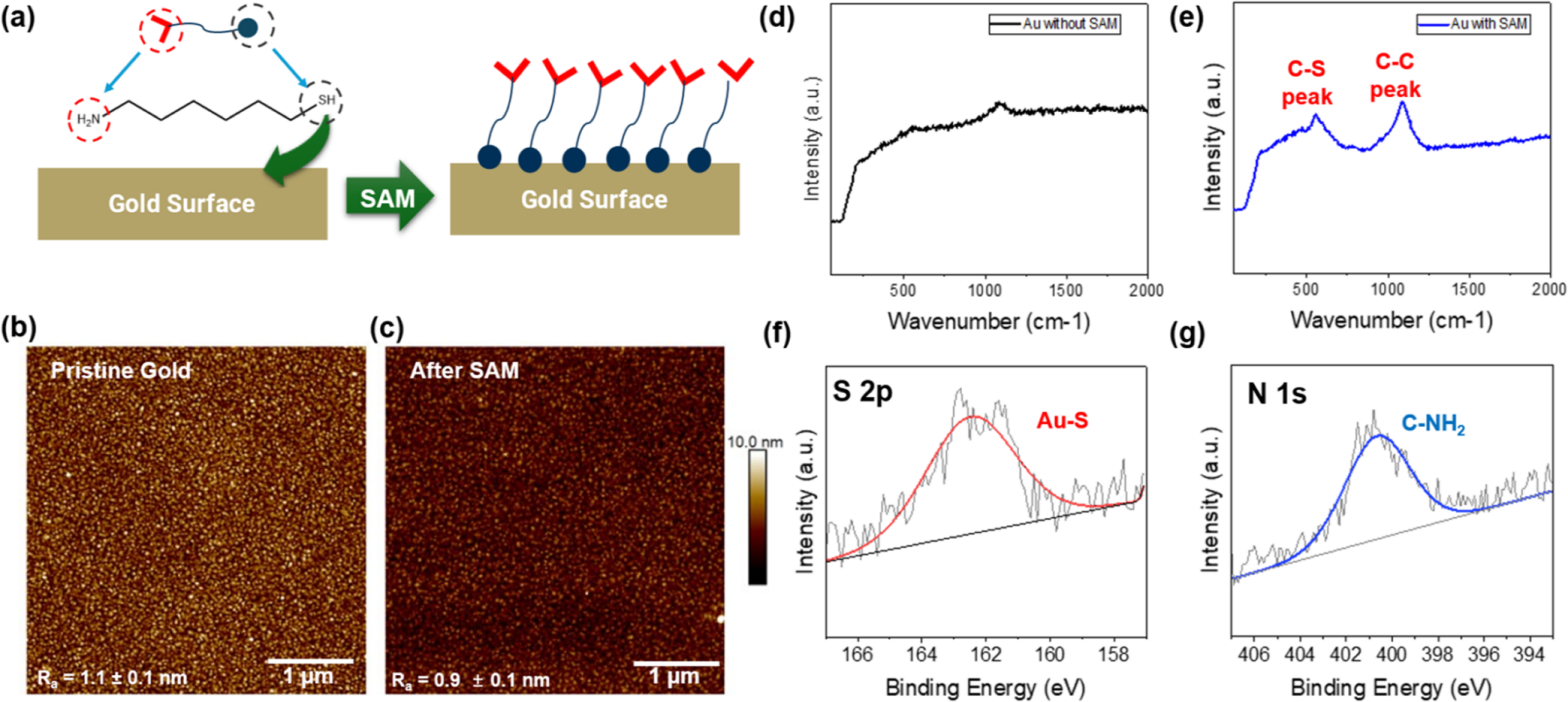
Characterization of amino-functionalized SAM on gold surfaces. (a) Schematic of SAM formation from amino-functionalized thiol on gold. AFM topographical image comparison (b) before and (c) after SAM treatment (color z-scale is 10 nm as displayed on the right side), showing a uniform SAM surface with very low microroughness. (d, e) Raman spectra with characteristic aromatic stretches. (f, g) XPS S 2p and N 1s binding energy spectra for the SAM-modified surface confirm Au-bound thiols and primary amines on the surface.

To confirm the successful SAM modification, atomic force microscopy (AFM) characterization was employed to directly validate the topographic features of the SAMs.^35–37^ Figure 6b presents the AFM topographic image of the pristine gold surface, displaying an average surface roughness (*R*_a_) value of 1.1 ± 0.1 nm for a 500 nm × 500 nm surface area, common for gold coatings. The grainy circular features visible in the AFM images, with approximate dimensions of around 30 nm, represent the typical polycrystalline grain structure of the sputtered gold substrate. The SAM-modified gold surface, which exhibits a uniform, densely packed monolayer with a low microroughness of *R*_a_ value of 0.9 ± 0.1 nm, confirms surface uniformity and demonstrates successful assembly with a slight surface smoothing effect similar to other reports (Figure 6c).^33,38^

Next, Raman spectroscopic analysis and X-ray photoelectron spectroscopy (XPS) studies were conducted to confirm the elemental composition of the SAM-modified gold surface. Figure 6d,e shows the baseline Raman spectrum of bare gold and following SAM modification, respectively, displaying prominent peaks appearing at approximately 500 and 1100 cm^–1^, corresponding to C–S and C–C peaks after SAM modification, confirming the successful assembly of thiol-anchored molecules on the gold surface.^39,40^ Simultaneously, Figure 6f displays the S 2p binding energy region, exhibiting the characteristic S peak at approximately 162 eV, confirming the presence of thiol-anchored sulfur atoms bound to the gold substrate. Figure 6g presents the N 1s binding energy region, showing a characteristic peak at approximately 401 eV, directly confirming the amine-terminated nature of the SAMs and the presence of primary amine groups on the surface.^41,42^ The combined Raman and XPS evidence confirms the formation of an amino-terminated monolayer.

Finally, by combining the activated gramine amide with the SAM-modified gold surface under the LiHMDS catalyst, we performed the transamidation reaction to achieve covalent immobilization of the activated gramine amide onto the SAM-modified gold surface and followed the surface topography changes after the transamidation reaction with AFM imaging (Figure 7).

**Figure 7:**
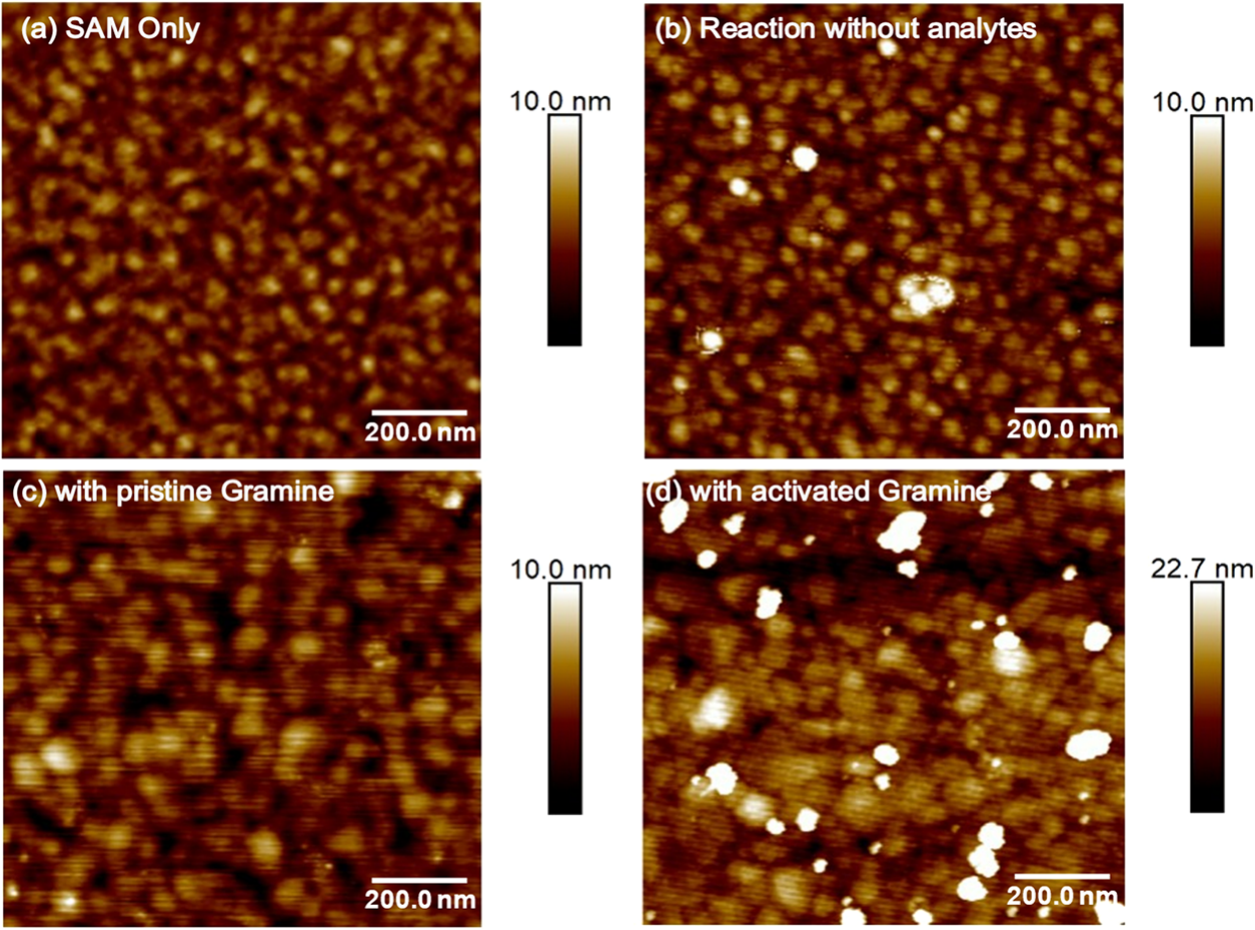
AFM characterization of selective gramine amide immobilization via transamidation (color z-scales shown on the right).  AFM topographical images: (a) Initial SAM-modified surface; (b) surface after reaction without adding analytes, (c) after attempting a transamidation reaction with pristine gramine without activation, and (d) after reaction with activated gramine amide. Also note the significant scale increase for the last image, implying an increment in microroughness.

Figure 7a shows the initial SAM-modified gold surface with a uniform topology and low surface roughness. When this surface was exposed to a LiHMDS catalyst-containing transamidation solution, without any analytes (Figure 7b) or with only pristine gramine without activation (Figure 7c), no significant morphological changes could be observed except for minor accumulations. In contrast, exposure to activated gramine amide resulted in dramatic surface changes (Figure 7d). The surface exhibited substantially increased microroughness, with densely packed molecular aggregates distributed across the entire scan area. These features are quite distinct even when the images are presented using a significantly enlarged z-scale range, indicating robust surface immobilization of activated gramine molecules. The phase image corresponding to each height image and the large-area height images are consistent with composition changes (Figures S10, S11). This notable change directly indicates the surface immobilization of target organic analytes to the SAM-modified surface, and the measured microroughness and apparent modulus values are further discussed below.

Based on these AFM images and complementary quantitative nanomechanical mapping (QNM) combined with ellipsometry and XPS measurements, quantitative surface analysis was performed to obtain statistical evidence for selective transamidation reactivity (Figure 8).^43,44^ First, the microroughness values obtained from the AFM height images were thoroughly compared for each step, with the average microroughness (*R*_a_) obtained by measuring multiple surface areas of 500 nm × 500 nm, avoiding accumulated particle sites (Figure 8a).

**Figure 8:**
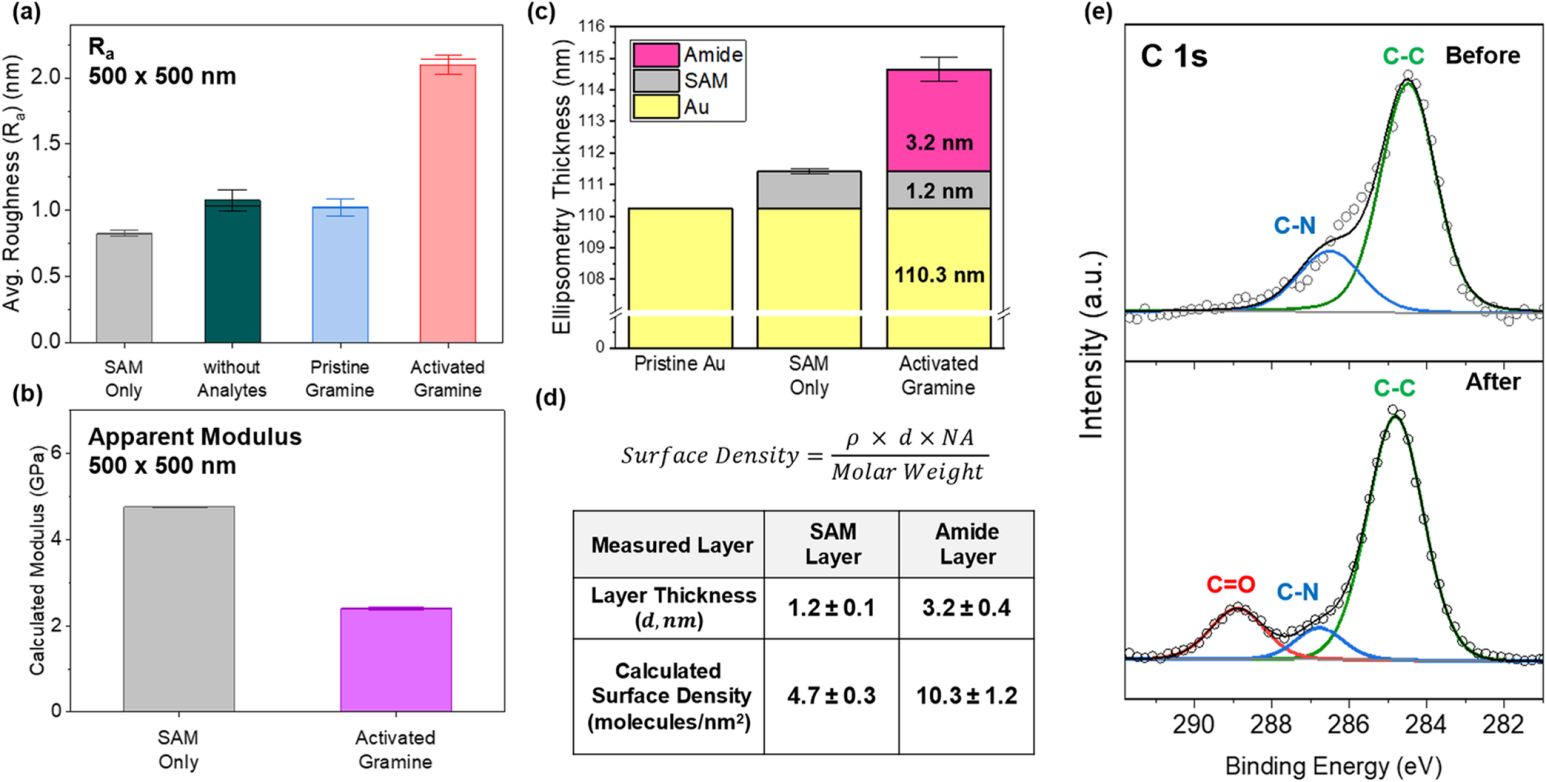
Quantitative characterization of gramine amide immobilization on SAM-modified gold surfaces. (a) Microroughness analysis demonstrating selective surface integration, inducing an average roughness change from as-activated gramine is anchored via transamidation, while the same procedure with the LiHMDS catalyst only or with pristine gramine shows minimal change. (b) QNM analysis showing a reduction in the apparent surface modulus after gramine amide immobilization. This decrease is consistent with the addition of soft organic material on the surface, supporting successful immobilization. (c) Layer thicknesses of pristine SAMs on gold and the anchored amide layer after the reaction were obtained by ellipsometry. (d) Calculated molecular grafting densities from ellipsometry data (equation shown: ρ = density, *d* = thickness, NA = Avogadro’s number). (e) XPS C 1s spectra showing emergence of a C=O peak after transamidation, confirming covalent amide bond formation.

This analysis shows that the pristine initial SAM-modified surface exhibited a low surface microroughness, as discussed previously, establishing a baseline for the well-ordered initial surface and densely assembled monolayer. The reaction with the LiHMDS transamidation solution alone, without adding analytes or only with pristine gramine without activation, increased the roughness only marginally to 1.1 ± 0.2 and 1.0 ± 0.2 nm, demonstrating minimal surface binding in the absence of reactive analytes. However, when activated gramine amide was introduced in solution, the surface microroughness increased 2-fold to 2.1 ± 0.2 nm, indicating the increasing surface adsorption of additional components, transforming the surface from a smooth surface into a rougher, heterogeneous surface appearance with adsorbed organic molecular aggregates in large quantities.

Moreover, QNM analysis revealed changes in surface mechanical properties following the immobilization process (Figures 8b and S12).^45^ The Derjaguin–Muller–Toporov modulus (DMT modulus) mapping of the pristine SAM surface showed average values of 4.8 ± 0.1 GPa. The same surfaces after transamidation and immobilization of activated gramine amide possessed a much lower modulus of 2.4 ± 0.1 GPa. This reduction in the apparent modulus is consistent with the formation of a softer, more compliant organic overlayer of immobilized gramine molecules and their aggregates on the surface. These measurements reflect the composite mechanical response of the thin organic layer and underlying substrate, as is typical for QNM analysis of ultrathin films on stiff substrates.^46^

Next, ellipsometry measurements of the pristine Au surface, SAM-modified surface, and SAM-modified surface after the transamidation reaction with gramine amide showed the effective thickness of absorbed organic material components (Figure 8c).^47^ The measured effective thickness of the assembled SAM layer was found to be 1.2 ± 0.1 nm, in accordance with existing literature.^48^ In contrast, the thickness of the SAMs with the adsorbed gramine amide layer increased manifold to 3.2 ± 0.4 nm layer due to the binding of additional drug molecules (Figure 8c).

Correspondingly, the surface grafting density was calculated from the layer thicknesses (Figure 8d).^49,50^ For example, by assuming a common density value for similar molecules of 0.9 g/cm^2^ for 6-amino-1-hexanethiol, our SAM molecule, a surface grafting density value of 4.7 molecules/nm^2^ can be obtained. This grafting density is close to the molecular cross-section area of thiols^51^ (reported values of 4 to 6 molecules/nm^2^ for well-defined SAM layers on Au), reconfirming the uniform, densely packed structure with a near-vertical orientation of thiol alkane molecules.^51,52^ On the other hand, the calculated surface grafting density of the amide layer showed a value of 10.3 molecules/nm^2^, which indicates further adsorption of gramine amides to the surface anchoring of double-layered aggregates. These calculations, based upon spectroscopic ellipsometry measurements, align well with AFM microroughness measurements that show a related increase in the nonuniformity of the surface morphology.^53^

Finally, XPS spectra of the C 1s region before and after the transamidation reaction showed further evidence for successful transamidation-mediated surface functionalization from the chemical side (Figure 8e). The C 1s spectra revealed significant chemical changes that occurred upon gramine amide immobilization. Before the reaction, the initial SAM surface demonstrated a single C-C peak predominantly at 284 eV, with a smaller C–N contribution at approximately 286 eV, characteristic of the well-ordered alkanethiol monolayer with minimal nitrogen-containing functionality.^54^ However, following transamidation and immobilization of activated gramine amide, a distinctive C=O peak emerged prominently at 289 eV, which is clearly visible in the lower part of the spectrum. It is important to note that the appearance of the carbonyl peak in XPS spectra was particularly significant, as it directly reflected the formation of the amide bond between the surface-bound carboxyl functionality and the gramine nitrogen nucleophile during the transamidation reaction.^55^ The large contribution of this new peak indicates a high surface coverage of immobilized gramine derivatives and effective surface anchoring via strong covalent binding.

Overall, we can conclude that the persistent C–C peak remained dominant in the XPS spectra, confirming the integrity of the underlying SAM-modified substrate after induced surface adsorption, while the new strong C=O contribution demonstrates the robust covalent grafting of activated gramine molecules onto the chemically modified gold surface.

## Conclusion

In conclusion, we demonstrated an integrated strategy combining Polonovski oxidation, transition-metal-free transamidation, and SAM surface engineering to selectively activate and immobilize tertiary amine biomarkers such as gramine, an important class of psychedelic biomarkers, onto modified gold surfaces. This approach overcame the fundamental challenge of tertiary amine electrochemical inertness, enabling the potential electrochemical detection of previously inaccessible biomarkers. Surface immobilization via the transamidation of biomarkers facilitated high selectivity and efficiency. Strong and selective covalent surface binding allows for direct analysis of the bonded molecules via surface-induced electrochemical signal changes suitable for biorecognition applications in prospective biosensing platforms, as will be further explored in future research.

Overall, this surface reactivity activating methodology, focused on designing selective surface reactivity, established a versatile platform for converting chemically inert tertiary amine biomarkers into surface-confined recognition elements suitable for selective FET-based electronic biosensing. The reactivity-triggering and selective binding approach suggested here is broadly applicable to tertiary amine-containing molecules, a class of organic molecules and biomarkers of neurochemical and forensic significance, opening new opportunities for label-free, high-sensitivity detection of structurally complex biomarkers previously inaccessible through conventional electronic sensory systems.

## Experimental Methods

### Materials

Gramine (≥97.5%, Sigma-Aldrich), tryptamine (≥97%, Sigma-Aldrich), hydrogen peroxide (30% v/v in water, Fisher Scientific), toluene (anhydrous, Sigma-Aldrich), acetone (HPLC grade, Fisher Scientific), acetic anhydride (Ac_2_O, ACS reagent grade, ≥98.0%, Sigma-Aldrich), dimethyl sulfoxide (DMSO, spectroscopic grade, Sigma-Aldrich), 6-amino-1-hexanethiol hydrochloride (Sigma-Aldrich), and lithium hexamethyldisilazide (LiHMDS, 1.0 M in Toluene, Sigma-Aldrich) were used as received without further purification. All reactions were performed at ambient temperature, unless otherwise specified.

### Conversion of Tertiary Amines to N-Oxides

The first step of chemical activation involved the selective oxidation of tertiary amines to their corresponding N-oxide intermediates using hydrogen peroxide as a mild and environmentally benign oxidant. In a typical procedure, the tertiary amine substrate (1 mmol) was dissolved in anhydrous acetone under ambient conditions. Hydrogen peroxide (1.2 equiv, 30% w/w aqueous solution or equivalent molar amount) was added dropwise to the stirred solution. The reaction mixture was maintained at room temperature and stirred for 24 h to ensure complete conversion. Upon completion, the reaction mixture was subjected to rotary evaporation under reduced pressure to remove acetone, and the crude N-oxide product was recovered by precipitation through centrifugation. The precipitate was collected and purified by washing with minimal acetone three times to remove residual starting material and organic impurities, followed by drying under a vacuum.

### Conversion of N-Oxides to Amides

The prepared N-oxide intermediate (1 mmol) was transferred to a clean, dry reaction vessel and dissolved in anhydrous dimethyl sulfoxide (DMSO) under a nitrogen atmosphere. Acetic anhydride (Ac_2_O, 1.2 equiv) was added in a single portion to the stirred solution. The reaction mixture was maintained at room temperature and stirred for 5 h. Upon completion, the reaction mixture was cooled to room temperature and diluted with distilled water to quench excess acetic anhydride. The aqueous mixture was extracted with ethyl acetate to transfer the organic product into the organic phase, and the combined organic extracts were washed at least three times with distilled water to remove DMSO and water-soluble byproducts. The organic layer was collected and subjected to rotary evaporation under reduced pressure to remove the ethyl acetate solvent.

### Fabrication of SAM-Modified Gold Surface

Gold electrodes (100 nm thick) were fabricated on glass substrates by using e-beam evaporation with a shadow mask patterning technique. Prior to functionalization, the gold surfaces underwent UV/ozone cleaning for 20 min. Subsequently, the substrates were submerged in an ethanolic solution containing 6-amino-1-hexanethiol (5 mM, Sigma-Aldrich) for approximately 18 h to create a SAM with functional primary amine (−NH2) groups. Following SAM formation, the functionalized substrates were rinsed at least three times with ethanol and deionized water.

### Transamidation Coupling of Activated Amides to the SAM-Modified Surface

The activated amides were dissolved in anhydrous toluene at a concentration of 1 mM, and the SAM-modified gold electrode was submerged in the solution under continuous stirring. Subsequently, LiHMDS (1.0 equiv, 1.0 M in toluene) was then added to activate the surface amine groups. The reaction mixture was stirred at room temperature for 15 h. Upon completion, the electrode was washed three times with toluene to remove unreacted reagents and physisorbed molecules.

### Characterization

^1^H NMR spectroscopy was performed by using a Bruker AV3 400 MHz spectrometer. All samples were dissolved in deuterated DMSO (DMSO-*d*_6_) at concentrations of 0.1 mg/mL and measured at room temperature. For N-oxide intermediates and Polonovski-activated amides, ^1^H NMR provided direct characterization of structural transformations with characteristic chemical shifts identifying the success of each synthetic step. Each sample was measured with a minimum of 32 scans to ensure an adequate signal-to-noise ratio. Data processing was performed using Bruker TopSpin and Mestrelab Mnova software.

Surface ζ-potential measurements were performed by using a Malvern Zetasizer Nano instrument equipped with a folded capillary cell (DTS1070). Samples were diluted in ultrapure water or other solvents and filtered through 0.45 μm syringe filters before being loaded into the DTS1070 cell. Measurements were conducted at 25 °C, with a minimum of three independent replicates per sample, each comprising 10 consecutive runs. ζ-potential values were determined from electrophoretic mobility data and expressed in millivolts (mV). Complementary solution conductivity was measured simultaneously with the same setup, while the molecular concentration of the samples was set to the same for comparative measurement.

FTIR analysis was conducted by using a Bruker Vertex 70 spectrometer equipped with an attenuated total reflectance (ATR) accessory. Sample solution containing purified gramine N-oxide and gramine amide was drop-casted on an ATR crystal, followed by thorough drying under vacuum for 24 h to remove any residual solvents. Measurements were performed in the wavenumber range of 4000–1200 cm^–1^, averaging 64 scans per sample to improve the signal-to-noise ratio. Background spectra were collected before each measurement and automatically subtracted from the sample spectra.

Raman spectra measurement was conducted using a WiTEC alpha300 R confocal Raman microscope to analyze the vibrational modes and oxidation state of the prepared samples. Prior to measurement, the samples were prepared by depositing the target material onto a glass substrate covered with aluminum foil to minimize background noise and enhance the signal clarity. A drop-casting method was employed for deposition, followed by controlled drying to achieve a uniform distribution. The microscope was operated with a 532 nm excitation laser, and spectral acquisition was performed by using a high-sensitivity CCD detector with an appropriate grating selection to resolve characteristic peaks with high precision.

Surface morphology and quantitative mechanical analysis were performed using a Bruker Dimension Icon microscope.^37,43^ The samples were prepared on a glass substrate cleaned with standard piranha solutions by the deposition methods described above, and the surface was washed multiple times using toluene and ethanol to remove impurities, followed by vacuum drying. Bruker RTESPA-300 tips with a nominal tip radius of 8 nm, spring constant of 40 N/m, and resonance frequency of ∼300 kHz were used, with measuring parameters of 512 to 1024 samples per line and a scanning rate of 0.5 to 1 Hz.

Scans were performed over 3.0 μm × 3.0 μm or other different-sized regions following standard procedures. Roughness measurements were conducted over at least three 500 nm × 500 nm areas chosen within the boundaries of individual flakes, accurately characterizing the surface without edge effects. For quantitative mechanical mapping, the probe-sample interactions measured with the PeakForce Tapping mode were translated into mechanical information about the sample. All AFM tips were similarly calibrated on a known material, sapphire, chosen for its hardness and lack of deformation during the tip–sample interaction, with a sharp tip before each sample. Ultimately, the indentation depth and load force were used for the mechanical response evaluation. Given the ultrathin nature of the SAM (∼1 nm) and organic adlayers (∼3 nm) relative to typical AFM indentation depths, the reported modulus values represent apparent composite mechanical responses of the organic layer and underlying Au substrate and should be considered semiquantitative indicators (we call it apparent modulus) of relative surface mechanical properties rather than absolute material moduli. Postprocessing and analysis, including image flattening, height measurements, cross-sectional height analysis, and microroughness calculations, were performed by using NanoScope Analysis 2.0 software.

XPS measurements were performed by using a Thermo Scientific K-Alpha spectrometer. SAM-modified Au samples fabricated on a glass substrate were dried in a vacuum for at least 3 h before analysis to remove any residual peaks. Survey scan spectra were taken two times with binding energies ranging from 0 to 1350 eV in 1 eV increments, and the high-resolution scans were collected ten times for each element in 0.1 eV steps. All spectra were calibrated by using the C 1s peak at 284.8 eV as a reference, and the obtained spectra were analyzed with Thermo Scientific Avantage Software.

Ellipsometry measurements were conducted by a Woollam M-2000U spectroscopic ellipsometer at different angles of 65, 70, and 75 degrees. The VWASE software was used to determine the thicknesses of SAM and SAM-amide layers deposited on Au-coated glass substrates. The measurements were modeled using a multilayer stack consisting of the glass substrate, Au layer, and SAM or SAM-amide layers at ambient conditions. During model parameter fitting, the optical properties of the substrate and Au layer remained fixed, and the parameters associated with the SAM layers were varied for the data fit. Measurements were performed at more than three different locations on each sample, and the final thickness was determined from consistent results across these independent measurements.

## Supplementary Material



## Notes

Additional experimental data, including chemical structure, digital photographs, NMR, Conductivity, XPS, and AFM images of analytes, substrates, and modified surfaces (PDF)
