# Supplementary material for: Anchoring Stealth Amine Biomarkers via Coupled Chemical Activation for Surface Immobilization
Source: Langmuir. 2026 Apr 7;42(15):10364–74. doi: 10.1021/acs.langmuir.5c06581 (PMC13104157; doi:10.1021/acs.langmuir.5c06581)
Supplement: Supplementary file 1 [file la5c06581_si_001.pdf]

Supporting Information

Anchoring Stealth Amine Biomarkers via Coupled Chemical Activation for Surface Immobilization

*Jinyoung Choi, Sehyun Park, Jaejun Lee, Vladimir V. Tsukruk**

School of Materials Science and Engineering, Georgia Institute of Technology, Atlanta, Georgia 30332, United States

*Corresponding Author

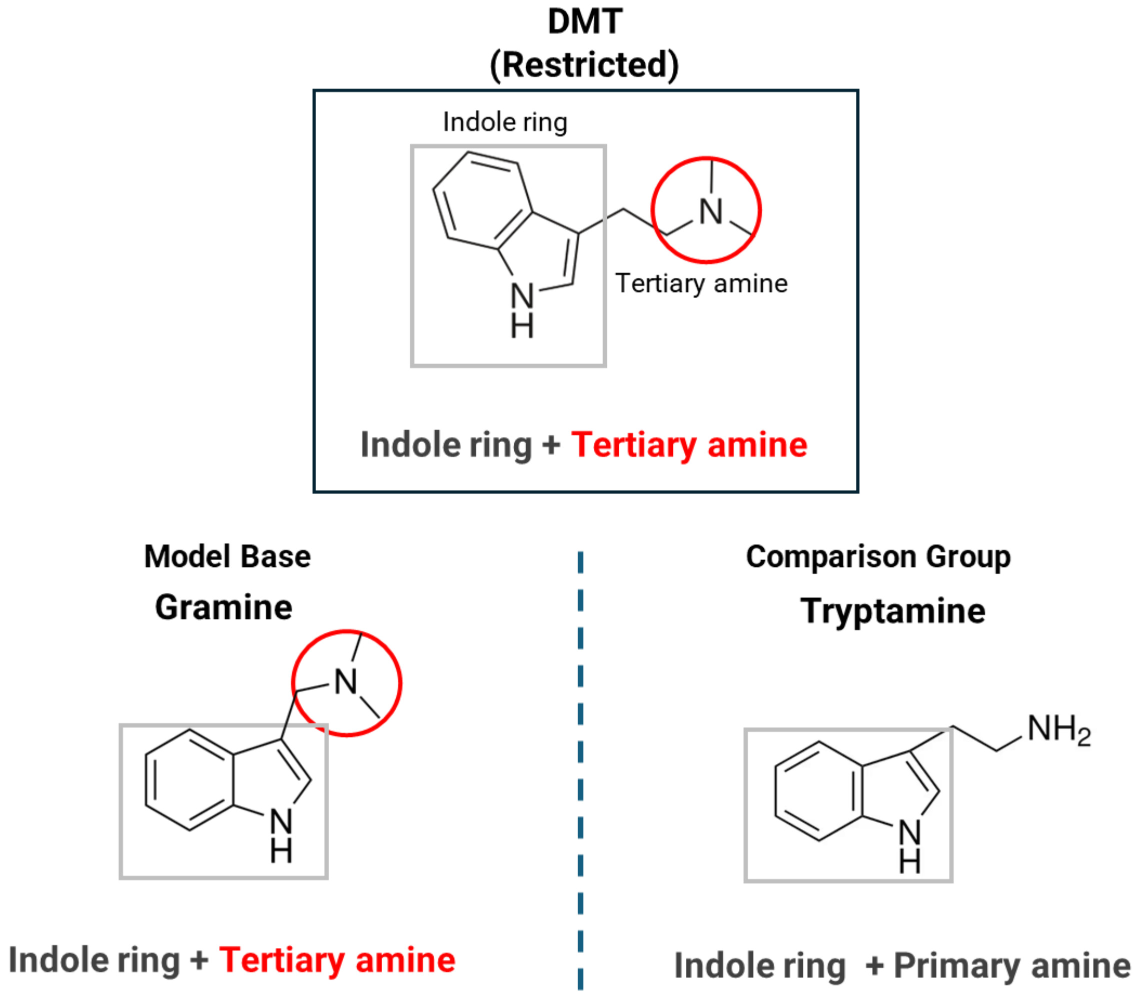

Figure S1 Chemical structure of targeted molecules used for experiments in this paper. Due to the drug restrictions on using DMT(upper) directly, we used gramine(left) with a similar structure, are model base, while tryptamine(right) with primary amines instead of a tertiary amine was used as a comparison group.

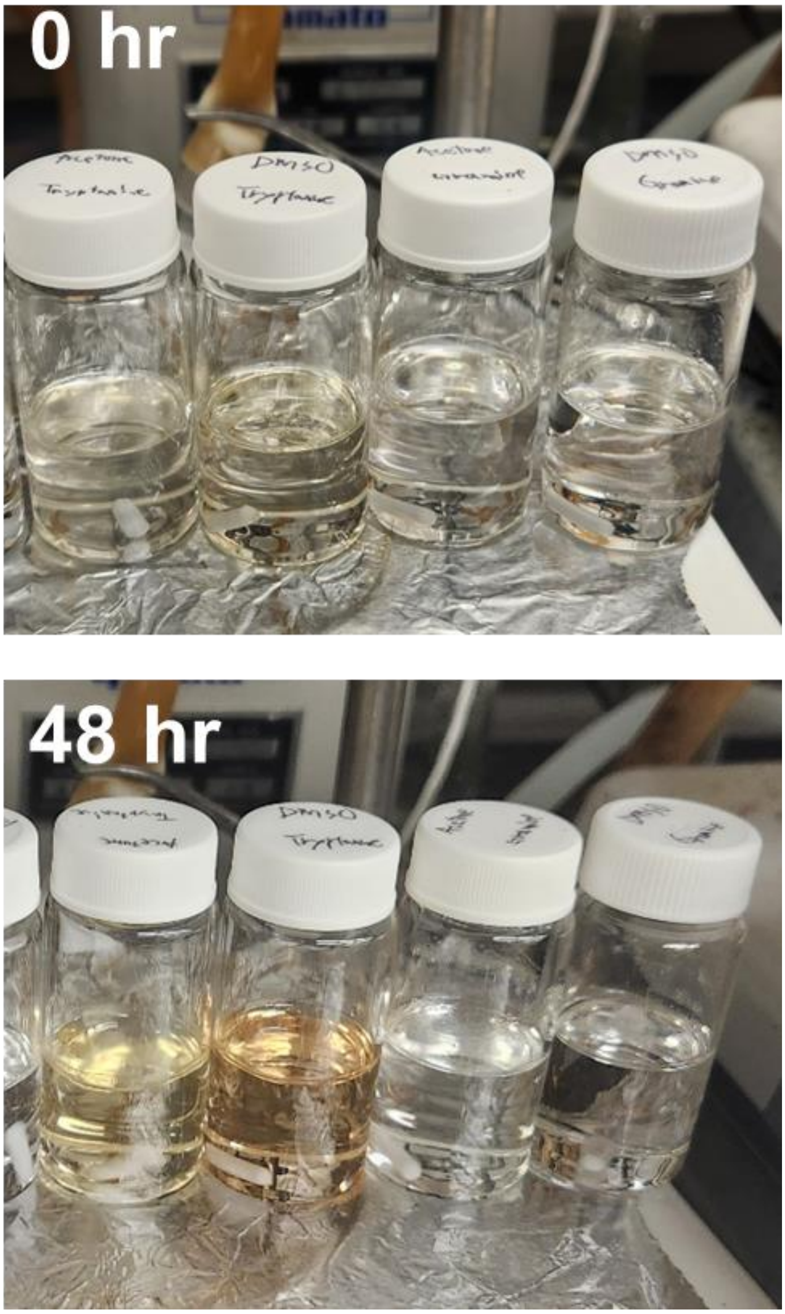

Figure S2 Photographs of tryptamine and gramine in reaction with H_2O_2, from the beginning (upper) to after 48 hours (below). From the left, each vials contain tryptamine in Acetone and DMSO, followed by gramine in the same solvent sequence.

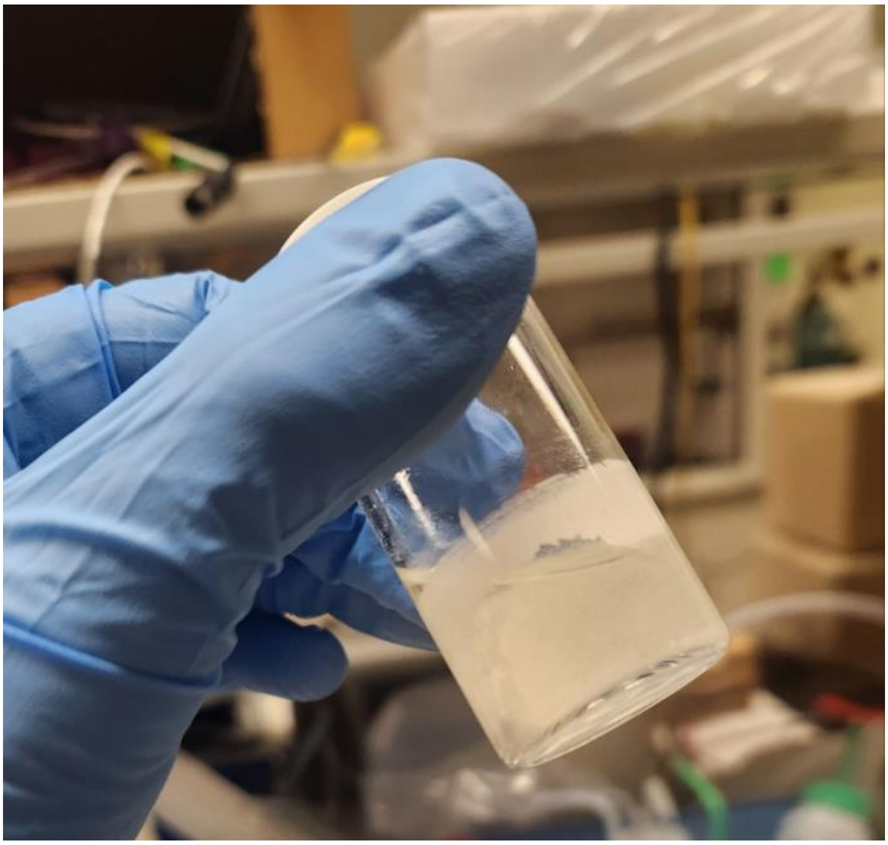

Figure S3 Digital photograph of Gramine N-Oxide after 1 week of reaction with H_2O_2 in acetone. The reduced solubility of N-oxide in acetone causes it to precipitate in white powders.

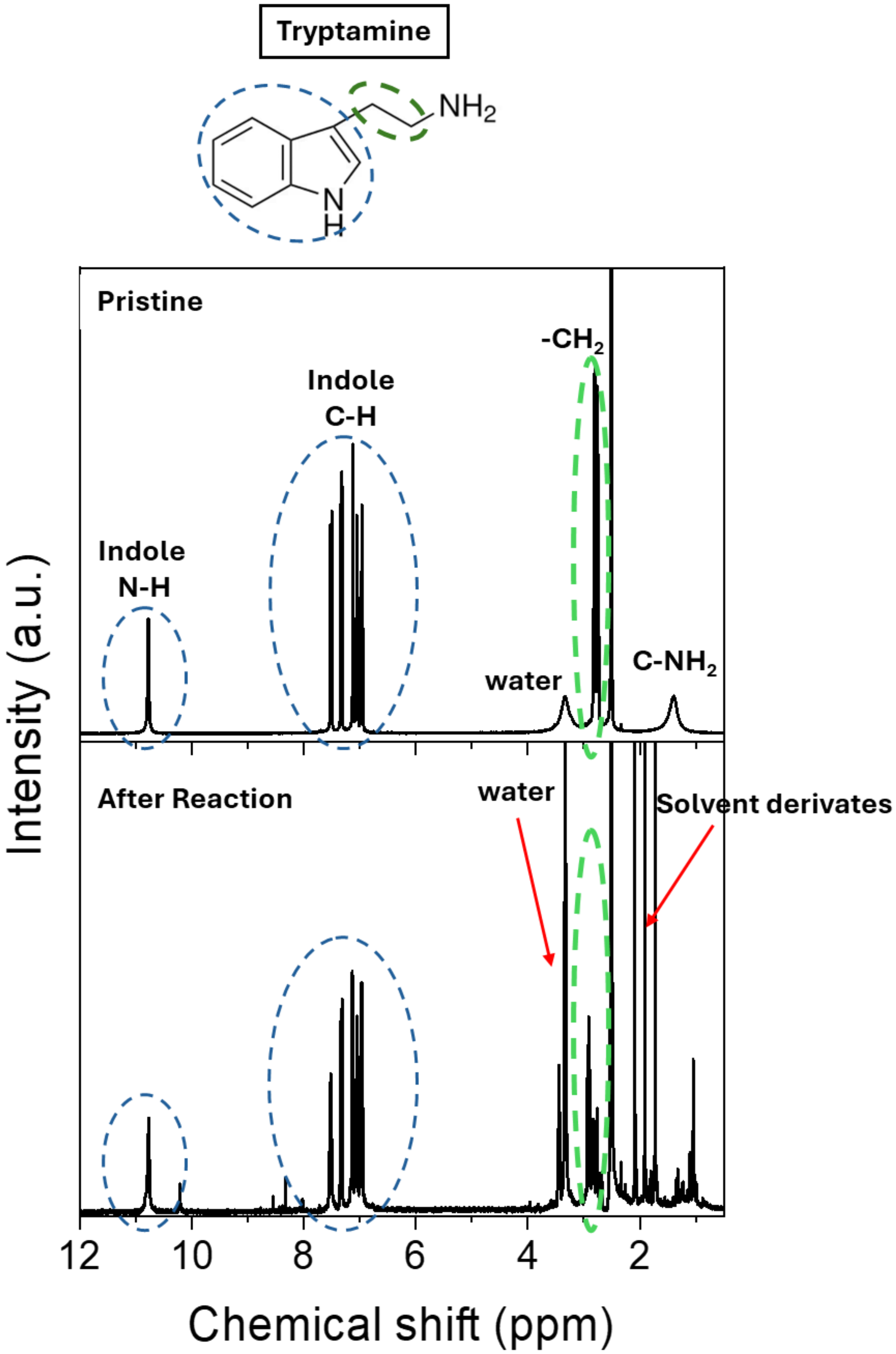

Figure S4 NMR spectra of tryptamine after exposure to H_2O_2. Unlike gramine, the tryptamine does not have a tertiary amine group that can be converted to N-Oxides, and the primary peak positions of the -CH_2 protons do not show a significant shift. Instead of reacting with the less-favored, controlled sample, the H_2O_2 formed different derivatives with solvents.

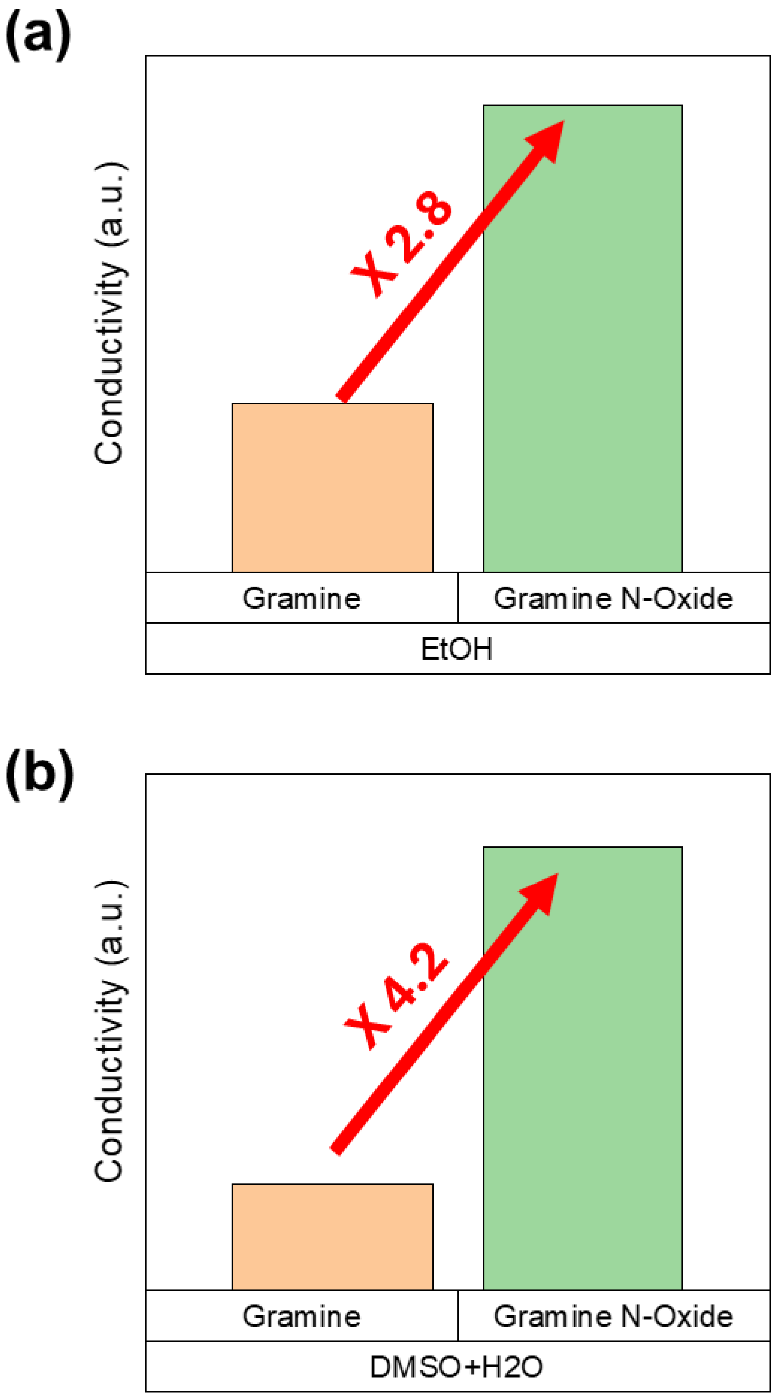

Figure S5 Solution conductivity of gramine and gramine N-oxide in (a) EtOH and (b) DMSO with water measured with the instrumentation described in the methods section.

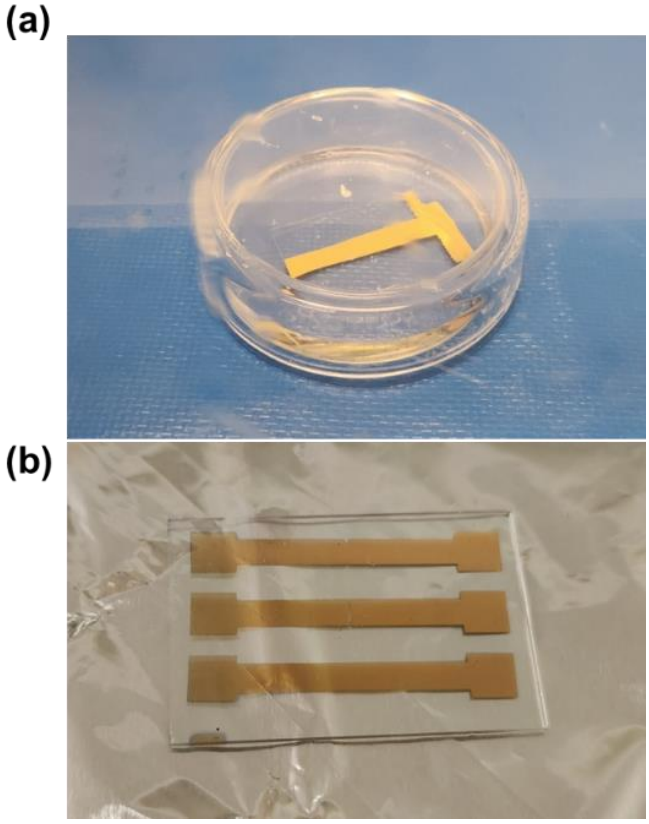

Figure S6 Digital photograph of Au substrates (a) undergoing SAM modification and (b) after modification. Substrates with sputtered gold with various shapes were fabricated for further utilization in FET devices.

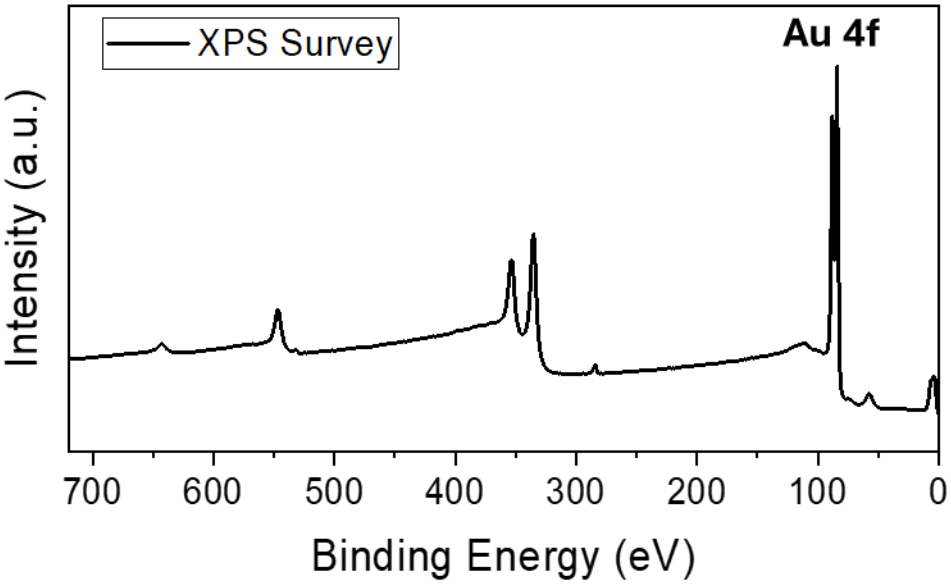

Figure S7 XPS survey spectra of the Au surface before SAM modification. The known Au 4f peak position is marked.

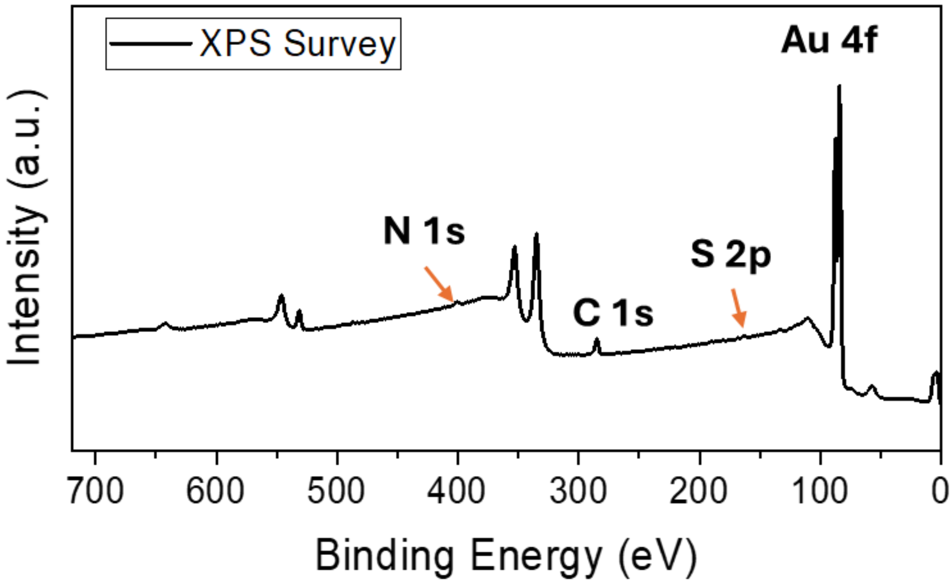

Figure S8 XPS survey spectra of the Au surface after SAM modification. Nitrogen and sulfur peaks appear as marked.

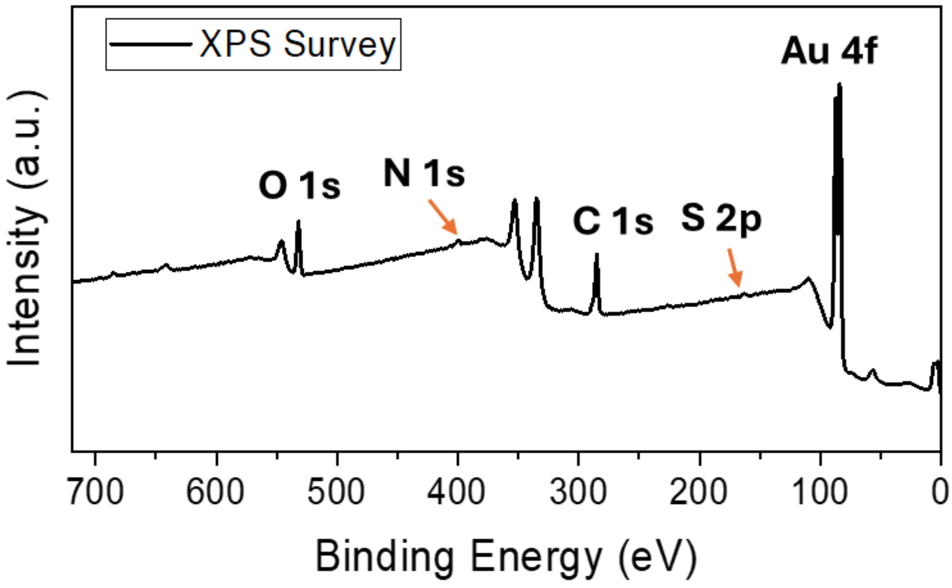

Figure S9 XPS survey spectra of SAM-modified Au surface after transamidation reaction with gramine amide. The O 1s peak becomes prominent due to the amide group addition.

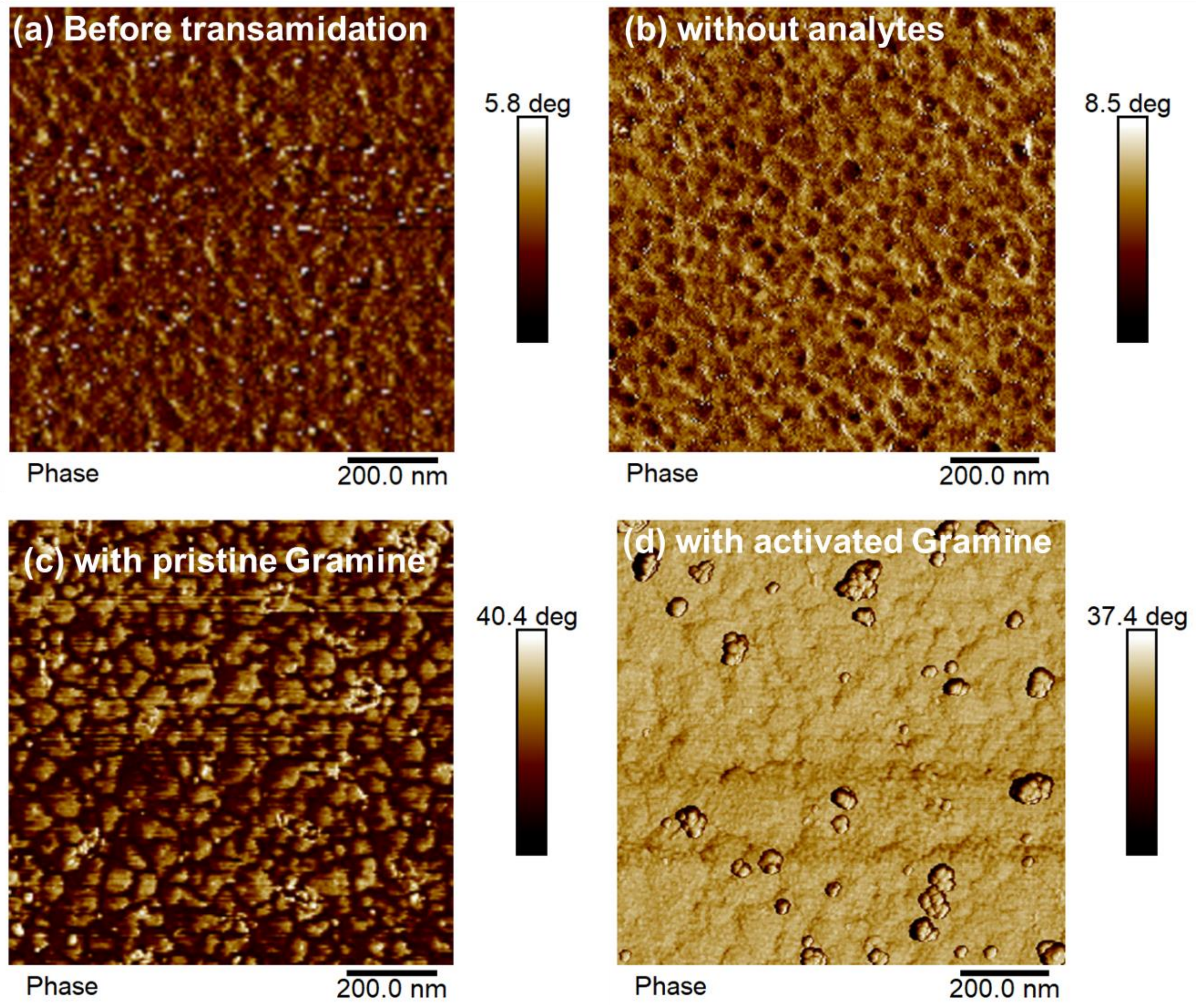

Figure S10 AFM phase images of SAM-modified gold surface: (a) before reaction, (b) after transamidation reaction attempt without analytes, (c) with pristine gramine, and (d) with activated gramine.

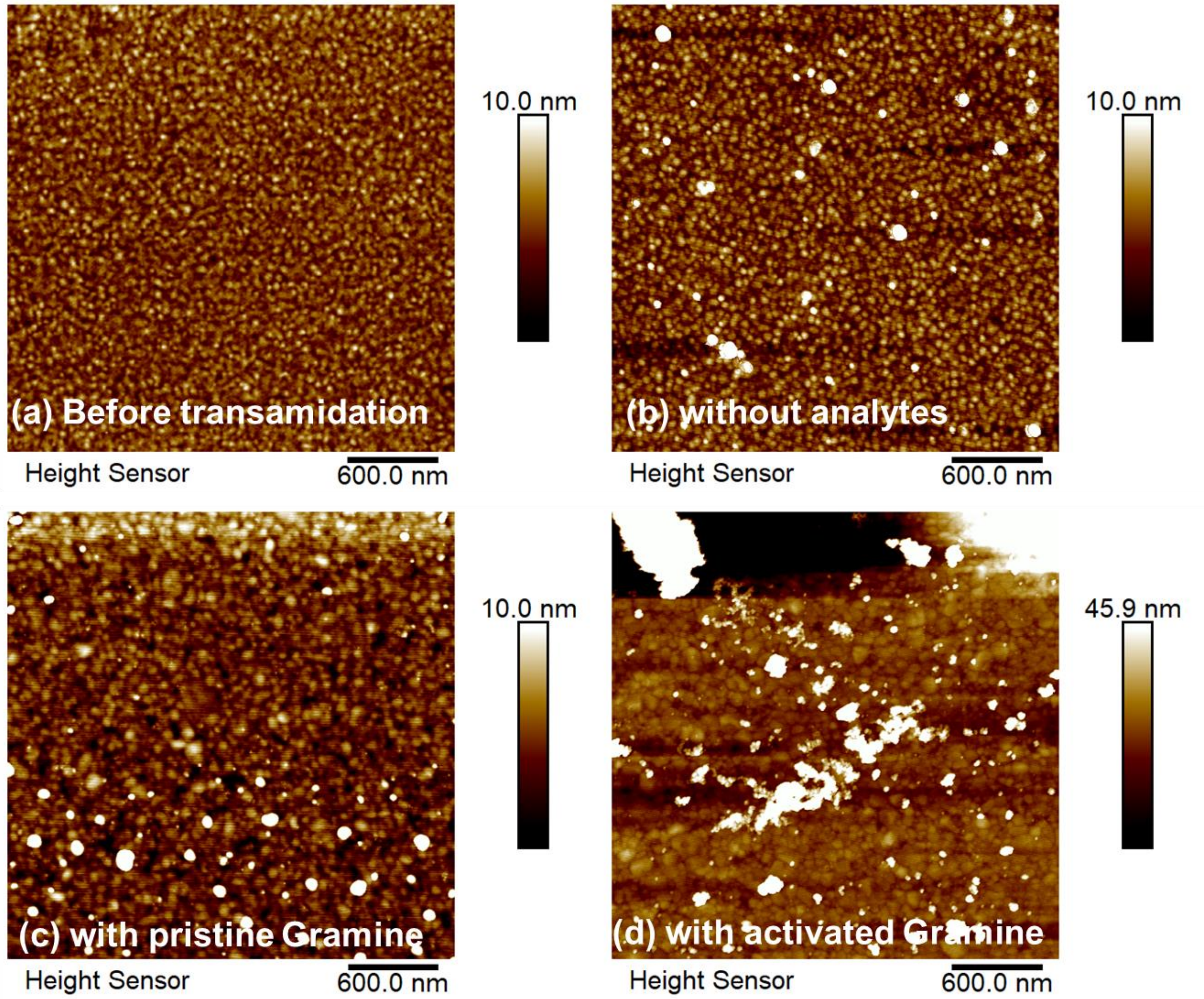

Figure S11 Large area (3um x 3um) AFM topographical images of SAM-modified gold surface: (a) before reaction, (b) after transamidation reaction attempt without analytes, (c) with pristine gramine, and (d) with activated gramine.

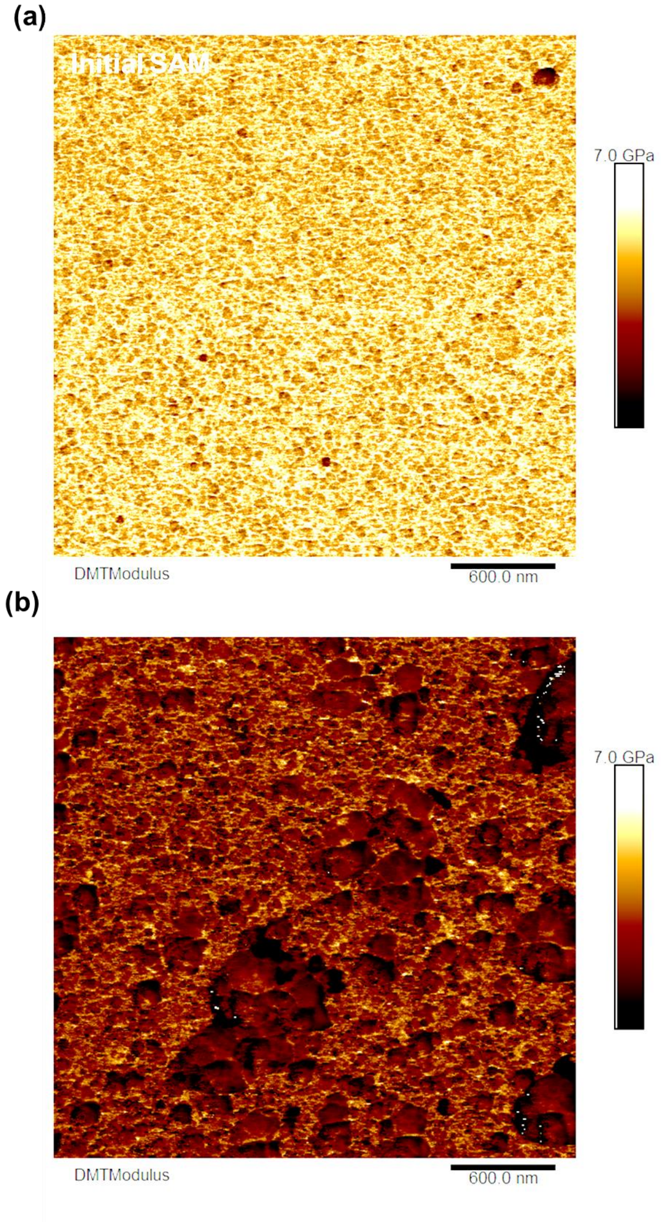

Figure S12 DMT modulus image of SAM-modified surface (a) before transamidation reaction and (b) after reaction with gramine amide with the same color z-scale. Significant overall reduction in average apparent modulus from 4.8 ± 0.1 GPa to 2.4 ± 0.1 GPa upon gramine amide immobilization, reflecting the formation of a softer organic overlayer, which can be directly observed by color change across all measured areas.
